# Comparative Metabolomic Analysis of *Dendrobium officinale* under Different Cultivation Substrates

**DOI:** 10.3390/metabo10080325

**Published:** 2020-08-10

**Authors:** Si-Min Zuo, Hai-Dong Yu, Weimin Zhang, Qiuping Zhong, Wenxue Chen, Weijun Chen, Yong-Huan Yun, Haiming Chen

**Affiliations:** 1 College of Food Science and Engineering, Hainan University, 58 Renmin Road, Haikou 570228, China; z285429797@163.com (S.-M.Z.); haidongyu@yeah.net (H.-D.Y.); zhwm1979@163.com (W.Z.); hainufood88@163.com (Q.Z.); hnchwx@vip.163.com (W.C.); chenwj@hainu.edu.cn (W.C.); 2 Institute of Environment and Plant Protection, Chinese Academy of Tropical Agricultural Sciences, Haikou 571101, China; 3 Engineering Research Center of Utilization of Tropical Polysaccharide Resources, Ministry of Education, Haikou 571101, China

**Keywords:** *Dendrobium officinale*, metabolomics, differential metabolites, cultivation substrates

## Abstract

*Dendrobium officinale*, a precious herbal medicine, has been used for a long time in Chinese history. The metabolites of *D. officinale*, regarded as its effective components to fight diseases, are significantly affected by cultivation substrates. In this study, ultra-performance liquid chromatography mass spectrometry (UPLC-MS/MS) was conducted to analyze *D. officinale* stems cultured in three different substrates: pine bark (PB), coconut coir (CC), and a pine bark: coconut coir 1:1 mix (PC). A total of 529 metabolites were identified. Multivariate statistical analysis methods were employed to analyze the difference in the content of metabolites extracted from different groups. By the criteria of variable importance in projection (VIP) value ≥1 and absolute log_2_ (fold change) ≥1, there were a total of 68, 51, and 57 metabolites, with significant differences in content across groups being filtrated out between PB and PC, PB and CC, and PC and CC, respectively. The comparisons among the three groups revealed that flavonoids were the metabolites that fluctuated most. The results suggested the *D. officinale* stems from the PB group possessed a higher flavonoid content. Kyoto Encyclopedia of Genes and Genomes (KEGG) pathway enrichment analysis indicated that the significantly regulated metabolites were mainly connected with flavonoid biosynthesis. A comprehensive profile of the metabolic differentiation of *D. officinale* planted in different substrates was provided, which supports the selection of an optimum cultivation substrate for a higher biomass yield of *D. officinale*.

## 1. Introduction

*Dendrobium* is a genus of valuable medicinal herbs in the family Orchidacea [1], containing 74 species and two varieties in China [2] of which many species can be used as medicine [3,4]. *Dendrobium officinale* is a precious species and famously known as one of the nine immortal herbs [5], as it possesses many kinds of physiological functions [6], including immune modulation [7], antitumor activity [8], and helping to reduce levels of blood glucose [9,10]. However, wild *D. officinale* is scarce because it requires a peculiar growth environment and takes a long time to grow. Moreover, wild *D. officinale* has been exploited excessively due to the large demand from the market [11]. Recently, in order to satisfy market demand and save wild resources, researchers tried to plant *D. officinale* by means of artificial cultivation [12,13]. The methods, which can be effectively applied to plant *D. officinale*, included a cultivation technique aiming to imitate the wild conditions and a plastic tunnel culture (PL). The PL technique in particular can simulate the growth environment of *D. officinale* in a greenhouse [14]. Indeed, the yield substantially increased as a result. However, the quality of artificial *D. officinale* is evaluated not only by the yield but also by the effective ingredients. It is widely accepted that ingredients, such as polysaccharose, flavones, and phenolic acids [15,16,17] are the main resource causing the benefits derived from *D. officinale*, and all of them fluctuate when the growth environment (cultivation substrate, illumination time, and temperature) of *D. officinale* changes. Thus, it is important to determine the optimal growth environment for *D. officinale* to gain a higher output and higher quality.

Over recent years, many research studies have been carried out about evaluating the quality of *D. officinale*. Some [18,19] used growth indices consisting of survival rate, plant height, stem diameter, and number of roots and leaves, which can represent the yield of the plants to some degree. A series of studies [20,21] was carried out using effective ingredients, such as polysaccharides, alkaloids, and total flavonoids, which can represent the quality of the *D. officinale*, to estimate the quality of the medicinal plant.

However, the efficacy of traditional Chinese medicine should be derived from the combination of several components rather than a single ingredient, such as polysaccharides, alkaloids, or flavonoids. Thus, the study of the detailed metabolite profile of a medicine sample, which could be used to guide the planting of the medicine, plays an important role in comprehensively evaluating medicinal efficacy. In this study, a widely targeted metabolomics analysis [22], integrating the “universality” of a nontargeted metabolomic and the “accuracy” of a targeted metabolomics [23], was employed to study the detailed metabolite profile of *D. officinale*. The widely targeted metabolomics analysis was implemented using liquid chromatography mass spectrometry (LC-MS), gas chromatography mass spectrometry (GC-MS), and nuclear magnetic resonance (NMR), combined with multiple reaction monitoring (MRM) [24]. The whole process involved conducting the instruments to detect the contents of small molecule metabolites in cells, tissues, and organs or organisms in different conditions; using multivariate statistical analysis to screen the differential metabolites, and employing pathway analysis of differential metabolites and revealing the physiological mechanisms behind changes.

Stems were collected from *D. officinale* cultivated in three kinds of cultivation substrates. By means of the widely targeted metabolomics analysis, ultra-performance liquid chromatography mass spectrometry (UPLC-MS/MS) was employed to obtain data (including retention time and peak area) from the metabolites of *D. officinale* for quantitative and qualitative analysis. The data obtained by quantitative and qualitative analysis were further analyzed using multivariate statistical analysis to observe whether the difference of the metabolites was significant, removing the interfering signals, and screening the differential metabolites of *D. officinale* in three different substrates. Finally, metabolic pathway analysis was conducted to search the metabolic pathway involved. The results revealed the difference of *D. officinale* metabolism among the three cultivation substrates (pine bark (PB), coconut coir (CC), and a pine bark: coconut coir 1:1 mix (PC)) and could provide basic information to guide *D. officinale* planting.

## 2. Results

### 2.1. Data Evaluation

To assess the repeatability of samples and stability of instruments, Pearson’s correlation coefficients were computed with the “cor” function in R (Ver. 3.5.0). The results of correlation analysis are given in Table 1. Correlation coefficients between intragroup samples (PB vs. PB, CC vs. CC, PC vs. PC) were higher than intergroup samples (PB vs. PC, PB vs. CC, PC vs. CC), which illustrated that the reproductivity of samples and the stability of the instrument were good. Thus, the method was suitable to carry out qualitative and quantitative analysis. The comparisons of PB vs. PC, PB vs. CC, and PC vs. CC exhibited a high correlation coefficient (0.8638, 0.9078, and 0.9108, respectively), showing that the metabolites screened in the three pairs of comparative groups were reliable.

### 2.2. Widely Targeted Metabolomics Profiling Results

In this study, the widely targeted metabolomics technique was used to detect the metabolic information of *D. officinale* from three substrates—PB, PC, and CC. Qualitative and quantitative analysis were carried out on the data obtained from UPLC-MS/MS. The total ion current (TIC) maps (Figure S1) of mass-spectrometry analysis of quality control (QC) samples detected at different times were well-overlapped, showing the instrument had good stability. As results, 529 metabolites were detected in total, and they were divided into 12 classes including flavonoids, phenolic acids, lipids, nucleotides and derivatives, alkaloids, amino acids and derivatives, lignans and coumarins, organic acids, quinones, tannins, terpenoids, and others. Among them, the top three most represented metabolites were flavonoids, phenolic acids, and lipids, which contained 135, 75, and 72 metabolites, respectively. After annotating all metabolites to the Kyoto Encyclopedia of Genes and Genomes (KEGG) compound database, 203 metabolites were matched, among which there were 116 metabolites with significant differences.

### 2.3. Principal Component Analysis

**Figure 1:**
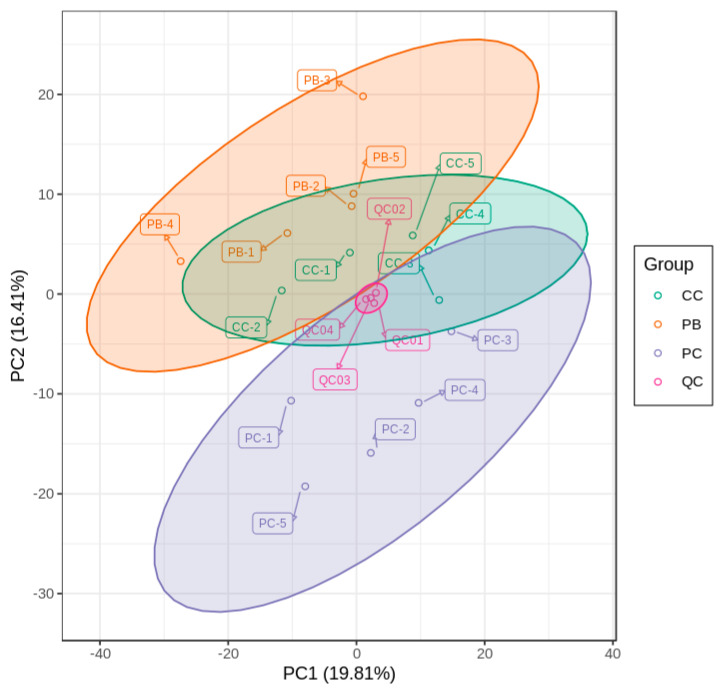
Score plot of principal component analysis (PCA) for *D. officinale* samples planted in pine bark (PB), coconut coir (CC), pine bark: coconut coir 1:1 mix (PC), and quality control (QC) samples. PC1 represents the first principal component and PC2 represents the second principal component.

The metabolic distribution trend among different groups and the degree of discreteness of different molecules in each group are shown in Figure 1. QC samples were gathered in a group and separated from other samples, which shows the QC samples had good repeatability. The samples from PB and PC were separated in the direction of PC2, and those from PC and CC were also separated in the direction of PC2. However, the samples from CC and PB overlapped a lot not only in the direction of PC1 but PC2 as well. The overall results of the PCA indicate the samples of PC were different compared with the samples of PB and CC, while the difference between PB and CC was not significant.

### 2.4. Orthogonal Partial Least Squares Discriminant Analysis

**Figure 2:**
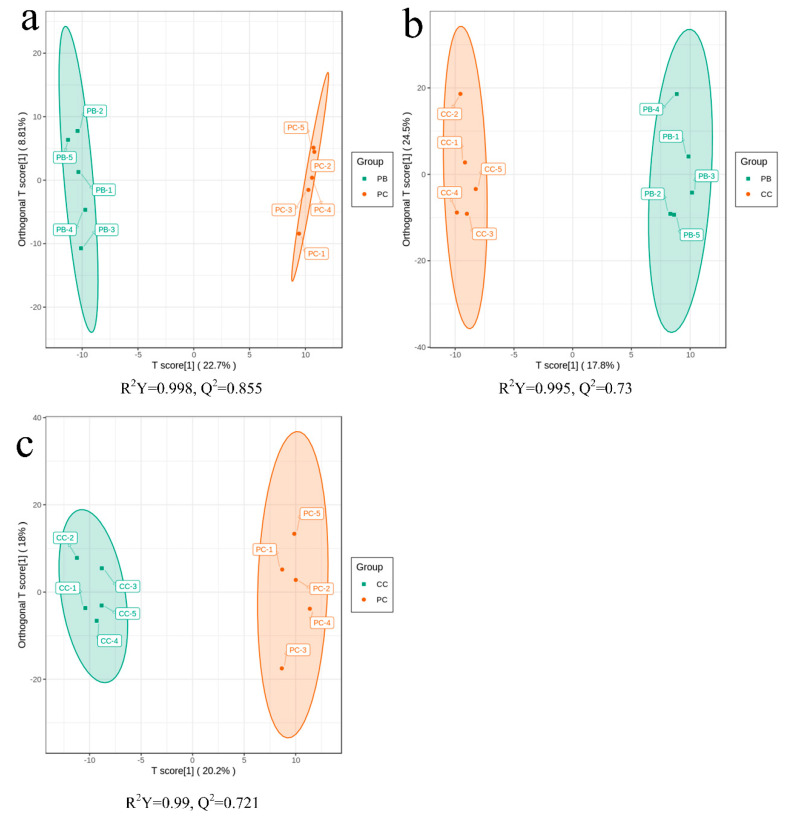
Score plots of orthogonal partial least squares discriminant analysis (OPLS-DA): (**a**) PB vs. PC; (**b**) PB vs. CC; (**c**) PC vs. CC. R^2^Y is the interpretation rate of the model to the **Y** matrix (Class label) and Q^2^ the predictive ability of the model. The model is valid when Q^2^ > 0.5. The *X*-axis is the predictive principal component, and the *Y*-axis is the orthogonal principal component.

Principal component analysis (PCA) can extract the main information effectively, but it is not sensitive enough to exclude the variables with smaller correlations; orthogonal partial least squares discriminant analysis (OPLS-DA), however, can achieve this. The variables with significant differences could also be screened by OPLS-DA. The OPLS-DA score plots are shown in Figure 2. Samples from the groups of PB vs. PC, PB vs. CC, and CC vs. PC had an obvious degree of separation, which illustrated that the metabolites generally exhibited significant differences among the *D. officinale* samples cultured in different substrates. To validate the OPLS-DA models, seven-fold cross-validation and 200 permutation tests [25] were conducted in this study (the results of permutation tests are shown in Figure S2). The R^2^ and Q^2^ values of the models of PB vs. PC, PB vs. CC, and PC vs. CC were 0.998 and 0.855, 0.995 and 0.73, 0.99 and 0.721, respectively, indicating that the models were of high fitness and predictability. Meanwhile, it can be said that the variable importance in projection (VIP) values generated from the OPLS-DA model were also reliable.

### 2.5. Identification of Differential Metabolites

Metabolites that differed significantly between *D. officinale* samples cultured in different substrates were screened using univariate analysis and multivariate statistical analysis. In this study, VIP values (VIP ≥1) combined with fold-change (FC) values (|log_2_ (FC)| ≥ 1) were used to screen these metabolites. In general, the metabolites with VIP values over 1 contributed a lot to the classification in the OPLS-DA model, and the FC value was used to screen metabolites with content that varied significantly by substrate.

**Figure 3:**
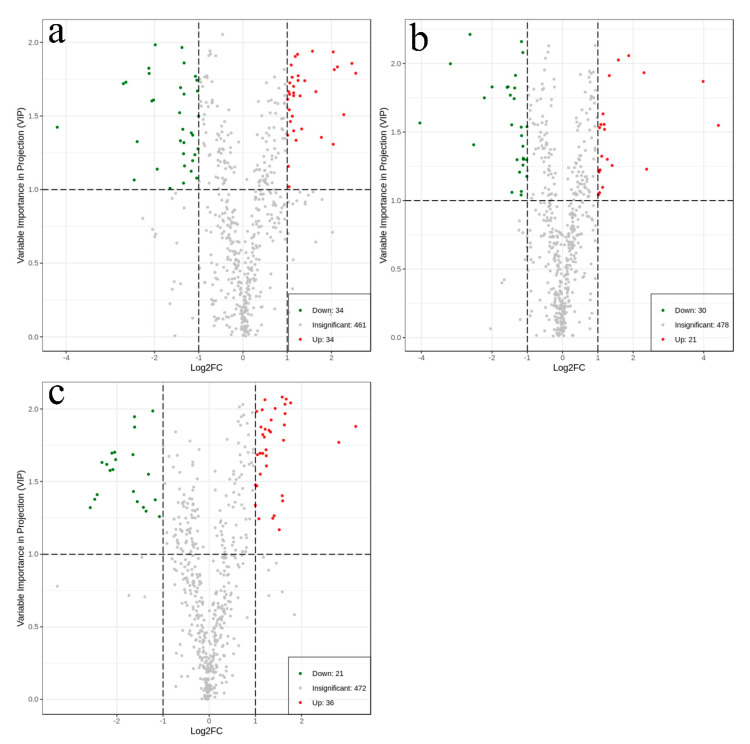
Volcano plots for (**a**) PB vs. PC; (**b**) PB vs. CC; (**c**) PC vs. CC. The green dots in the plots illustrated that the differential metabolites were significant and down-regulated, while the red dots illustrated that the differential metabolites were significant but up-regulated, and the black dots illustrated that the metabolites could be detected in samples but did not have any significant difference.

Volcano plots are shown in Figure 3 to display the metabolites that differed significantly. The detailed information of differential metabolites among the groups can be seen in Tables S2–S4.

From Figure 3a, it can be seen that there were 68 metabolites with significantly different content between the PB and PC groups. Compared with PB, PC contained 34 metabolites with an upregulated state and 34 metabolites with a downregulated state. A total of 51 metabolites significantly different in content between the PB and CC groups (Figure 3b), among which 21 metabolites increased and 30 metabolites decreased in content in the CC group compared to the PB group. A total of 57 metabolites were recognized as significantly different between PC and CC (Figure 3c). Among these metabolites, there were 36 metabolites with significantly higher content in PC than CC.

**Figure 4:**
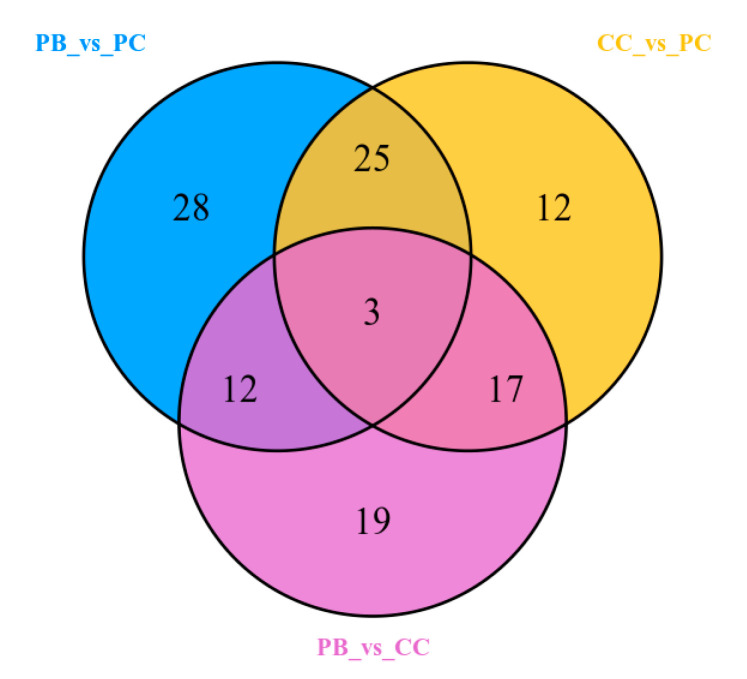
Venn diagram illustrating shared or unique metabolites that differed significantly in terms of content among the different comparison.

Based on the differential metabolites that had significantly different content in the comparisons of PB vs. PC, PB vs. CC, and PC vs. CC, Venn diagram analysis was conducted to display the metabolites that existed commonly or uniquely in the group. As shown in Figure 4, a total of 116 metabolites (Table S1) that existed in at least two groups were shown to obviously change in content. A further comparison for these metabolites’ contents among the three groups was conducted and the results are shown in Table 2. Among the 116 differential metabolites, there were 53 flavonoids, accounting for nearly 40 percent of all identified flavonoids, and these showed a great variation of content during growth in different substrates. Compared to the PB group, there were 21 out of 32 flavonoids down-regulated and 17 out of 21 flavonoids down-regulated in PC and CC, respectively. Besides flavonoids, 22 out of 72 lipids altered significantly, and compared with the PC group, the contents of most lipids (13 out of 16) decreased in PB, and nearly whole lipids (13 out of 14) decreased in PC. The distribution of all metabolites that differed significantly in content showed that flavonoids and lipids were the metabolites that fluctuated most.

**Figure 5:**
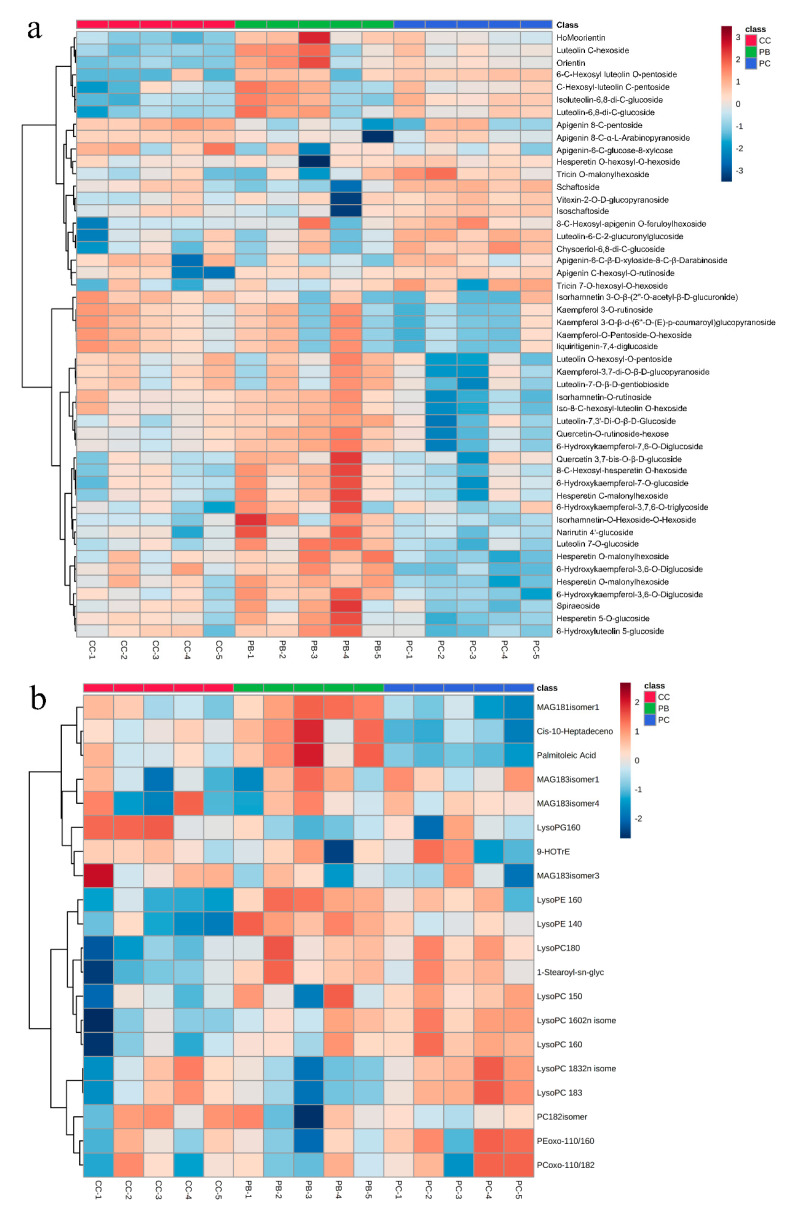
Heatmaps of hierarchical cluster analysis (HCA): (**a**) flavonoids; (**b**): lipids. The abscissa is used to display the names of samples, and the ordinate on the right is used to display the names of metabolites. The deeper the red color, the higher the content of the metabolites; the deeper the blue color, the lower the content of the metabolites.

Hierarchical cluster analysis (HCA) was conducted to display the trend of metabolic variation based on the differential metabolites of flavonoids and lipids, respectively. Heatmaps of flavonoids and lipids are shown in Figure 5. Figure 5a shows that more than two thirds of the flavonoids that differed significantly in content exhibited the highest contents in the group of PB when compared with PC and CC. Compared to the group of PC, the samples in the CC group possess a higher content for a majority of flavonoids. Similarly, from Figure 5b, we can see that a majority of lipids preferred accumulating in PB compared with PC and CC, while almost all of the lipids with significant variation in content had the lowest contents in CC. The above comparisons might suggest that the cultivation substrate of PB is more suitable for *D. officinale* to accumulate flavonoids and lipids. That is to say, PB may be the optimal cultivation substrate for *D. officinale*, with the highest biomass yield among PB, CC, and PC.

### 2.6. Pathway Enrichment Analysis of Differential Metabolites

**Figure 6:**
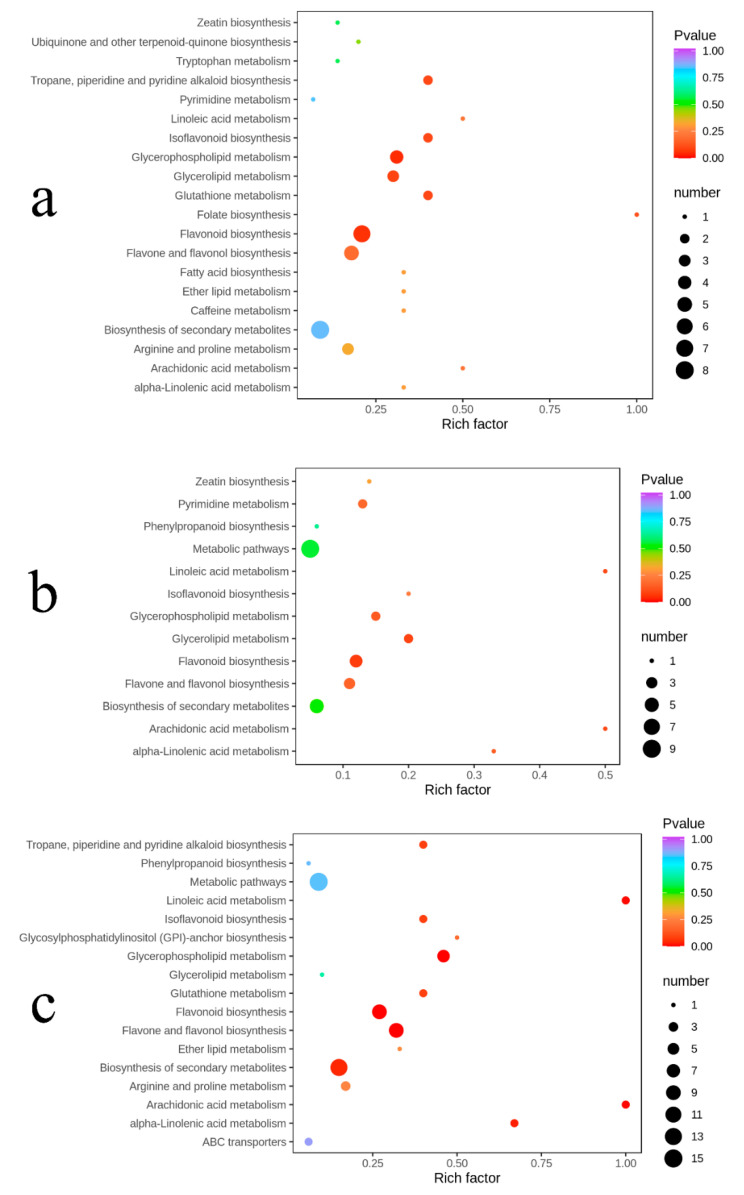
KEGG enrichment maps of differential metabolites: (**a**) PB vs. PC; (**b**) PB vs. CC; (**c**) PC vs. CC. The abscissa represents the enrichment factor of the pathway and the ordinate shows the names of pathways. The color of the dot represents the *p*-value, and the deeper the red of the dot, the stronger the enrichment effects. The size of points represents the number of metabolites enriched in the pathways.

A total of 203 metabolites in 543 identified metabolites were annotated in the KEGG compound database, of which only 17 metabolites showed significant differences in content in the comparisons of PB vs. PC, together with PB vs. CC and PC vs. CC. Furthermore, ten more differential metabolites, which have been detected in the samples but were not annotated on the KEGG pathway, were added to KEGG pathways according to their similar properties, such as chemical structures with known KEGG compounds. The results of the KEGG pathway enrichment analysis are shown in Figure 6.

The differential metabolites in the comparisons of PB vs. PC were annotated in 24 pathways, among which flavonoid biosynthesis and glycerophospholipid metabolism changed obviously. Similarly, the differential metabolites between PB and CC were annotated in 13 pathways, and the flavonoid biosynthesis and glycerolipid metabolism altered prominently. The differential metabolites between PC and CC were involved in 17 pathways, totally. Among them, the pathways, including flavonoid biosynthesis, flavone and flavonol biosynthesis, and glycerophospholipid metabolism, changed significantly (*p* < 0.01). In the comparisons between PB and PC, PB and PC, and PC and CC, only flavonoid biosynthesis was significantly distinct.

## 3. Discussion

*Dendrobium officinale* is a medicinal and edible plant of great economic value, exhibiting many physiological functions. However, most *D. officinale* on the market is planted artificially, and thus their quality is uneven. Here, we employed a widely targeted metabolomics technique to explore the metabolites with small molecules and evaluate the quality of *D. officinale* based on the contents of these metabolites.

In this study, the obvious separation among the three groups in PCA results indicated that the metabolism of *D. officinale* collected from different cultivation substrates differ a lot. The three cultivation substrates (PB, PC, and CC) have different physical properties (e.g., water-holding capacity) and chemical properties (e.g., content of nitrogen, potassium, and phosphorus). Therefore, it may be said that the difference of these properties in different cultivation substrates could affect the metabolism of *D. officinale* greatly. It has been reported [26] that the physical and chemical properties of soil have a definite effect on the contents of the primary medicinal components of *D. officinale* under four cultivation modes.

The metabolomic profiling results suggested that flavonoid compounds had maximum counts of metabolites in *D. officinale* stems. Flavonoids were also the metabolites with the greatest variation in the three cultivation substrates. For this reason, the flavonoids could be used to evaluate the quality of *D. officinale*. Flavonoids were also used to evaluate *D. officinale* quality based on medicinal effectiveness in a previous study [27], because they perform many physiological functions. The results of differential metabolites analysis of PB vs. PC, PB vs. CC, and PC vs. CC (detailed information of significantly differential flavonoids is shown in Tables S2–S4) suggested that PB was the optimal cultivation substrate. This supports a recommendation for employing certain cultivation substrates to increase the contents of some components. Our result is the same as a previous study [24] conducted using a widely targeted metabolomic analysis to reveal metabolic alterations of *Epimedium pubescens* leaves at different growth phases.

It should be noted that PB was only the optimal cultivation substrate when compared against PC and CC, and we only grew *D. officinale* in Haikou, China. Environmental factors have a great influence on *D. officinale* quality, so findings may not be generalizable.

## 4. Materials and Methods

### 4.1. Plant Samples and Chemicals

A base, located in Haikou Hainan, China, was suitable to plant *D. officinale* because of its mild climate and abundant rainfall. Five-month-old *D. officinale* plantlets from Zhejiang province were transplanted in November 2017 to a porous mesh basin with a size of 14.2 cm × 10.2 cm × 10.3 cm (length × width × height) in a greenhouse equipped with sunshade and sprinkling facilities. Each *D. officinale* plantlet was planted in a basin loaded with cultivation substrate to a volume of two thirds. The culture substrates included pine bark (PB), coconut coir (CC), and a pine bark: coconut coir 1:1 mix (PC), all of which had been sterilized and soaked in water. Each treatment replicated six units and every unit contained nine basins treated the same way. The plants were watered regularly, and the growth environment was monitored over time. *D. officinale* plantlets were shaded properly, cooled, and ventilated during summer and autumn. *D. officinale* stems were harvested almost two years later. The samples were packaged with a valve bag and then frozen using liquid nitrogen and finally place in storage at −80 °C.

All reagents were HPLC-grade. Methanol, acetonitrile, and ethanol were purchased from Merck (Darmstadt, Germany) and standard compounds of dimethyl sulfoxide (DMSO) were obtained from BioBioPha (Yunnan, China).

### 4.2. Sample Preparation and Extraction

Sample preparation and extraction were performed following reference [28]. Samples were freeze-dried in a lyophilizer (Scientz-100F, Xi Yu equipment Co. Ltd., Shanghai, China). Then, stems were ground with a zirconia bead for 1.5 min at 30 Hz using a mixer mill (MM 400, Retsch Technology, Haan, Germany). A total of 100 mg of powder was weighed and dissolved in aqueous methanol (0.6 mL, 70%) overnight at 4 °C. Then, the solution was whirled six times to improve the extraction rate. After centrifugation at 10,000× *g* for 10 min, the supernate was gained and filtrated employing the microfiltration membrane (SCAA-104, 0.22 μm pore size; ANPEL, Shanghai, China). The filtration was packed in a sample bottle for UPLC-MS/MS analysis. In addition, quality control (QC) samples were prepared by mixing three kinds of extractions from three different groups with the same weight.

### 4.3. UPLC-MS/MS Analysis

The prepared sample extraction was analyzed by means of a UPLC–ESI-MS/MS system, including a UPLC system (Shim-pack UFLC SHIMADZU CBM30A, Shimadzu, Kyoto, Japan) and a ESI-MS/MS system (Applied Biosystems 4500 Q TRAP, AB SCIEX, Foster City, CA, USA). The separation operation was implemented using a 1.8 μm C18 column (100 mm × 2.1 mm ID) (ACQUITY UPLC HSS T3, Waters, Wexford, Ireland) equilibrated at 40 °C. The mobile phases A and B were prepared by mixing ultrapure water and acetonitrile with 0.04% acetic acid, respectively. Chromatographic separation was completed by way of a gradient elution program: First, the mobile phase B rose from 5% to 95% within 10 min linearly. It was maintained at the level of 95% from 10 to 11 min, dropped to 5% from 11 to 11.1 min and held steady until 14 min. The temperature of the column oven was 40 °C, the injection volume was 4 μL, and the flow rate was 0.35 mL/min. The effluent was alternatively connected to an ESI–triple quadrupole-linear ion trap (QTRAP)-MS.

Mass-spectrometry detection was conducted on a triple quadrupole-linear ion trap mass spectrometer, API 4500 Q TRAP UPLC-MS/MS system equipped with an ESI Turbo Ion-Spray interface. The main parameters were as follows: The temperature of electrospray ionization was set to 550 °C; the voltages of ion sprays were 5500 V and −4500 V in positive and negative ion mode, respectively; the curtain gas (CUR) was 30 psi, and the collision-activated dissociation (CAD) was high [29]. Each ion pair was scanned at the conditions of the optimum declustering potential (DP) and collision energy (CE) in the triple quadruple pole (QQQ) system [30].

In the process of UPLC–ESI-MS/MS analysis, a QC sample was put behind each 10 test samples to monitor the stability of the instrument.

### 4.4. Qualitative and Quantitative Analysis of Metabolites

On the basis of the self-built database (MetWare database, MWDB [30,31]) of Maiteville Biotechnology Co., Ltd. (Wuhan, China) and the public secondary mass-spectrometry database (e.g., MassBank, KNApSAcK, HMDB [32], MoTo DB and METLIN [31]), qualitative analysis of metabolites was carried out. During analysis, interference, which came from isotope signals and repeated signals of K^+^, Na^+^, and NH_4_^+^ ions, as well as fragment ions derived from the metabolites which had higher molecular weight, was excluded by in-house software written in Perl.

The quantification of metabolites was completed by the MRM of triple quadrupole mass spectrometry [29]. In MRM mode, the precursor ions of the target substances were filtrated using a quadrupole rod to exclude the interference by removing the corresponding ions with different molecular weights. Next, many fragment ions, which were formed via the induced ionization in the collision chamber, were filtered by QQQ. A characteristic fragment ion was then selected to eliminate the interference of nongoal ions, rendering quantitative analysis more accurate and ensuring good repeatability. Finally, data gained from mass-spectrum analysis from different samples were used to calculate the integral results of the peak area. The retention time of the same kind of metabolite from different samples was calibrated by integral. The software Analyst 1.6.3 was used to process the mass-spectrometry data, and MultiaQuant was employed to complete the integration and calibration of chromatographic peaks.

### 4.5. Data Analysis

In this study, the data gained from the above stages were highly dimensional and needed to be simplified. Thus, PCA and OPLS-DA were utilized. Data analysis was conducted mainly using R [33] software. PCA analysis was performed by built-in “prcomp” function (Version 3.5.0, University of Auckland, Auckland, New Zealand), and data were processed by unit variance scaling (UV) to eliminate the influence of different measurement units before PCA. OPLS-DA was conducted on the raw data by the statistics function “MetaboAnalystR” [34] (Version 1.0.1, McGill University, Quebec, Canada). In this process, data were analyzed in two groups: experimental group and control group, encoded 0 and 1, respectively. They were transformed with log_2_ function and mean-centered. The VIP value was then extracted from the results of OPLS-DA. Finally, metabolites that exhibited statistically significant differences between each two groups of the three groups were screened by the conditions of VIP ≥1 and absolute log_2_ (FC) ≥1 [35]. Heatmap was conducted to exhibit the trend of the variation of differential metabolites by the pheatmap (Version 1.0.12) package of R software.

Authenticated metabolites were annotated based on the KEGG compound database [36] and annotated metabolites were then matched to the KEGG pathway database. Pathways that significantly regulated metabolites mapped to were fed into a metabolite sets enrichment analysis (MSEA), and their significance was calculated by the *p*-values from hypergeometric tests.

## 5. Conclusions

Quantitative differences were explored among *D. officinale* stems from three different substrates. The UPLC-MS/MS-based widely targeted metabolomics method was conducted to investigate the variation of the extracted metabolites. The results showed that the chemical constitution of *Dendrobium officinale* cultured in three substrates were equal, but the contents were significantly different from one class to another. Data were analyzed by multivariate statistical methods containing PCA and OPLS-DA. The results illustrated that 68, 51, and 56 differential metabolites were screened in PB vs. PC, PB vs. CC, and PC vs. CC, respectively. Further comparison with the HCA analysis for the 116 significantly differential metabolites revealed that flavonoid-type compounds were the main bioactive metabolites that were regulated obviously due to the variation of cultivation substrates, and the heatmap suggested that most flavonoids preferred to accumulate in the PB group. Consequently, it could be concluded that PB was the best cultivation substrate to improve the contents of flavonoids in this study. Furthermore, it is indicated that only “flavonoid biosynthesis” was significantly affected by the fluctuated metabolites in the KEGG pathway enrichment analysis.

## Figures and Tables

**Table 1:** Average Pearson’s correlation coefficients for comparisons of intragroup and intergroup samples.

Group	Average Pearson’s Correlation Coefficient
PB vs. PB	0.9403
PC vs. PC	0.9422
CC vs. CC	0.9519
PB vs. PC	0.8636
PB vs. CC	0.9078
PC vs. CC	0.9108

Note: the reproducibility of samples and the stability of the instruments are defined as good when the correlation coefficients of intragroup comparisons are higher than intergroup comparisons. The data were considered reliable when the correlation coefficients of intragroup samples were above 0.8.

**Table 2:** The number of metabolites that had significantly different contents in *D. officinale* cultured in PB, PC, and CC.

Group	PB vs. PC		PB vs. CC		PC vs. CC	
	Up	Down	Up	Down	Up	Down
Flavonoids (53/135)	11	21	4	17	12	11
Phenolic acid (16/75)	3	5	3	6	5	3
Lipids (22/72)	13	3	4	2	13	1
Amino acids and derivatives (3/64)	0	0	3	0	0	0
Alkaloids (7/41)	4	0	0	3	4	0
Nucleotides and derivatives (3/37)	1	1	2	0	0	0
Organic acids (3/34)	1	0	2	1	1	1
Lignans and Coumarins (6/17)	0	3	2	1	0	4
Quinones (1/2)	0	1	0	0	0	1
Others (2/49)	1	0	1	0	1	0
Significantly differential metabolites	34	34	21	30	36	21
Total metabolites that differed significantly in content (116)	68		51		57	

Note: the numbers in parentheses separated by (/) indicate the number of metabolites in that class that had significantly different contents across substrates metabolomic data. Up/Down represents the changed trend of the metabolite content in the comparison between the latter group and the former one.

## Supplementary Materials

The following are available online at https://www.mdpi.com/2218-1989/10/8/325/s1, Figure S1: The total ion chromatograms (TIC) of Dendrobium officinale QC samples: (a) positive ESI mode; (b) negative ESI mode., Figure S2: Plots of permutation tests on OPLS-DA models., Table S1: 116 differential metabolites screened by the group of PB vs PC, PB vs CC and PC vs CC, Table S2: Differential metabolites between PB and PC., Table S3: Differential metabolites between PB and CC., Table S4: Differential metabolites between PC and CC.
