# Supplementary material for: Comparative Metabolomic Analysis of Dendrobium officinale under Different Cultivation Substrates
Source: Metabolites. 2020 Aug 10;10(8):325. doi: 10.3390/metabo10080325 (PMC7465462; doi:10.3390/metabo10080325)
Supplement: Supplementary file 1 [file metabolites-10-00325-s001.pdf]

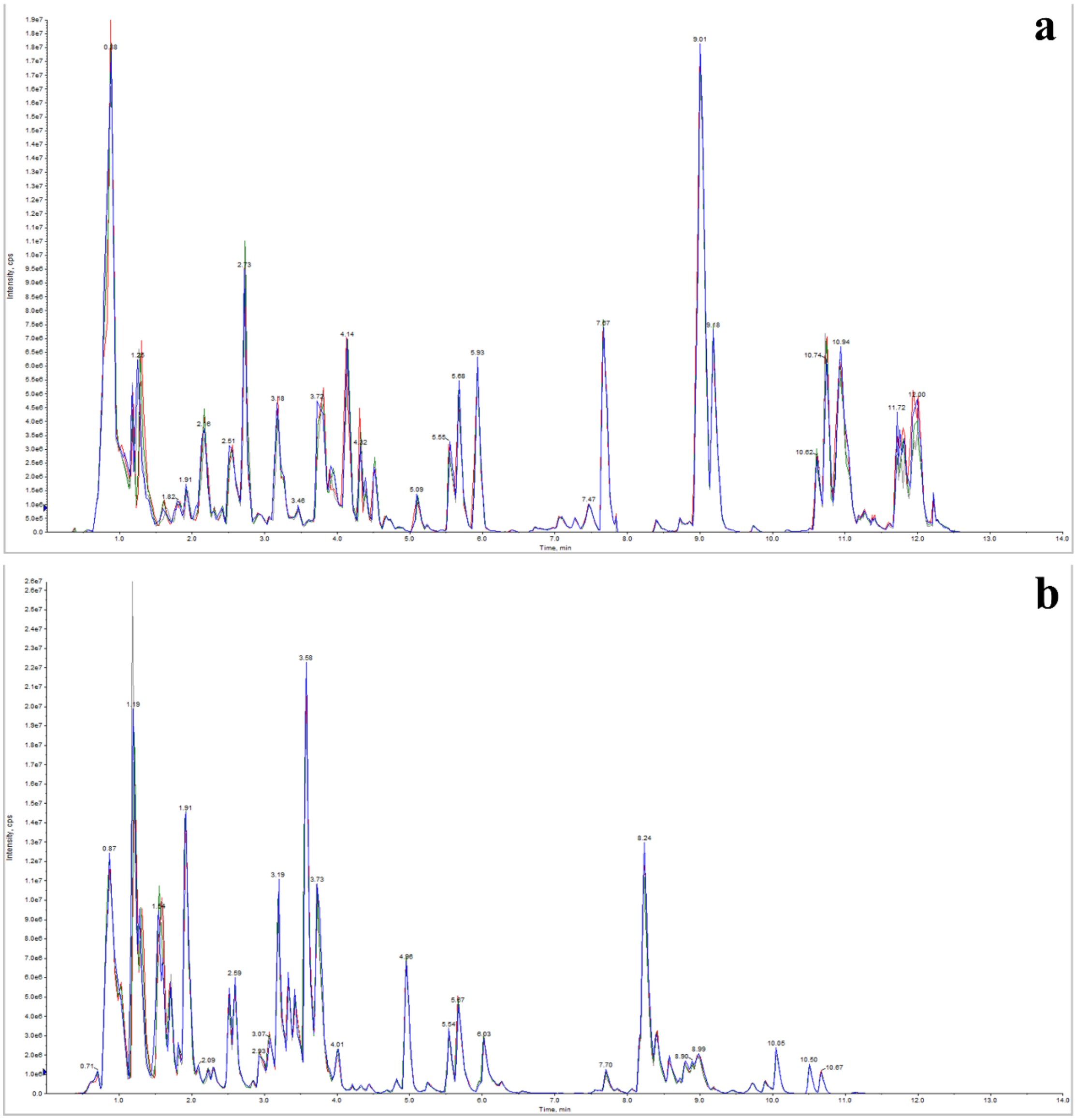

Figure S1 The total ion chromatograms (TIC) of *Dendrobium officinale* QC samples: (a) positive ESI mode; (b) negative ESI mode.

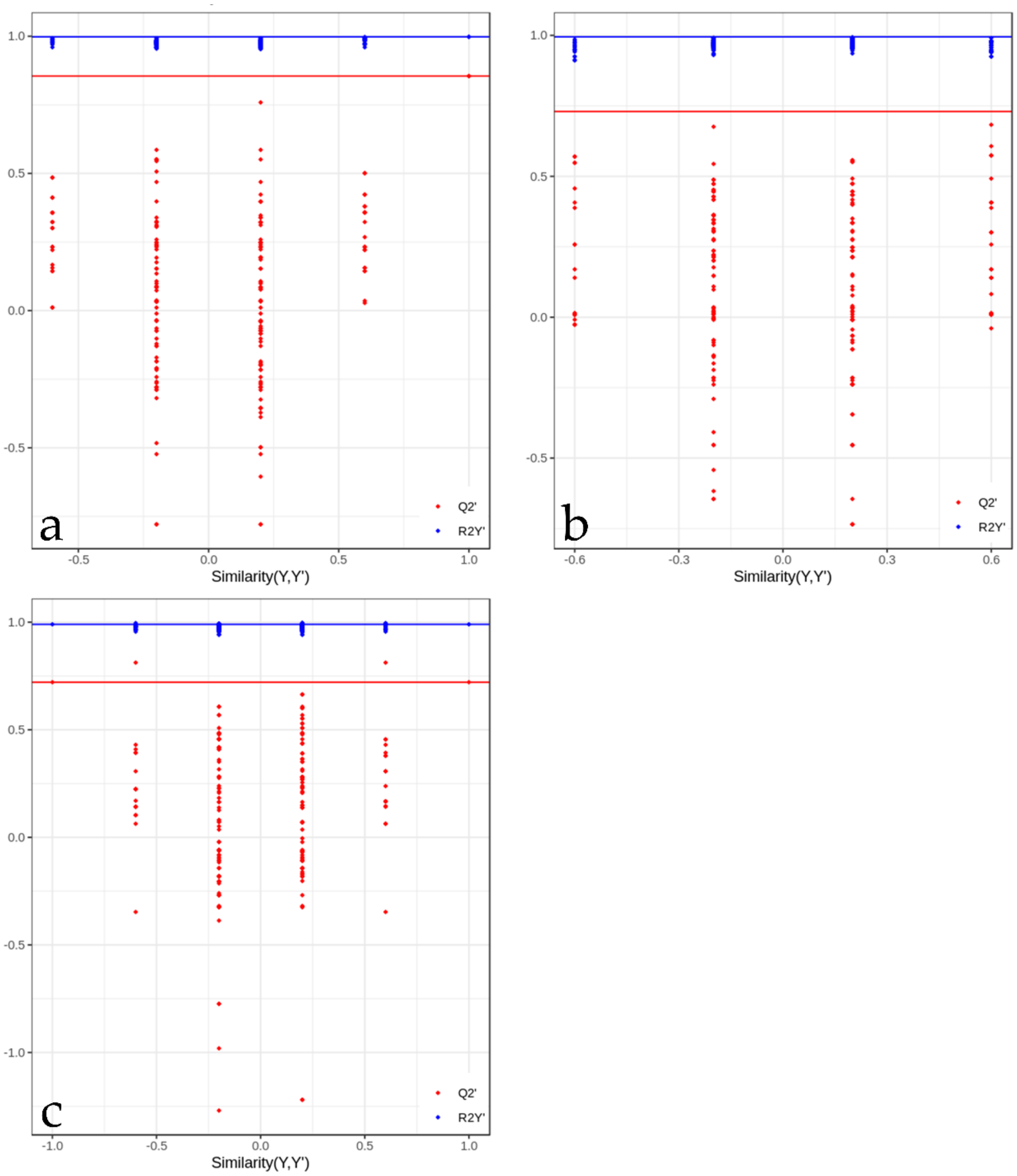

Figure S2 Plots of permutation tests on OPLS-DA models. The horizontal lines represented the parameters calculated in the original OPLS-DA model, the red and blue represented R2Y and Q2, respectively. The dots were the parameters calculated by permutation tests, the red dots were R^2Y' and the blue were $Q^{2\prime}$.

Table S1. 116 differential metabolites screened by the group of PB vs PC, PB vs CC and PC vs CC

No.	Compounds	Formula	Class
1	Isochrysoeriol C-hexosyl-O-hexoside	C28H32O16	Flavonoids
2	Iso-8-C-hexosyl-luteolin O-hexoside	C27H30O16	Flavonoids
3	Isoluteolin-6,8-di-C-glucoside	C27H30O16	Flavonoids
4	Isosinapic acid-hexoside	C17H22O10	Phenolic acids
5	Salicylic acid O-glycoside	C13H16O8	Phenolic acids
6	Pinoresinol-acetylglucose	C28H34O12	Lignans and Coumarins
7	6-Hydroxykaempferol-3,7,6-O-triglycoside	C33H40O22	Flavonoids
8	6-Hydroxykaempferol-7,6-O-Diglucoside	C27H30O17	Flavonoids
9	6-Hydroxykaempferol-3,6-O-Diglucoside	C27H30O17	Flavonoids
10	6-Hydroxykaempferol-7-O-glucoside	C21H20O12	Flavonoids
11	LysoPC(18:0)	C26H54NO7P	Lipids
12	PC(18:2)isomer	C26H51NO7P+	Lipids
13	Caffeoylcholine 6-glucoside	C20H30NO9+	Alkaloids
14	Hesperetin C-malonylhexoside	C26H28O13	Flavonoids
15	Apigenin-6-C-β-D-xyloside-8-C-β-Darabinoside	C25H26O13	Flavonoids
16	Vitexin-2-O-D-glucopyranoside	C27H30O15	Flavonoids
17	Quercetin 3,7-bis-O-β-D-glucoside	C27H30O17	Flavonoids
18	Luteolin-7,3'-Di-O-β-D-Glucoside	C27H30O16	Flavonoids
19	4-Caffeoylquinic acid	C16H18O9	Phenolic acids
20	liquiritigenin-7,4-diglucoside	C27H32O14	Flavonoids
21	Luteolin-6-C-2-glucuronylglucoside	C27H28O17	Flavonoids
22	Apigenin-6-C-glucose-8-xylcose	C26H28O14	Flavonoids
23	Chysoeriol-6,8-di-C-glucoside	C28H32O16	Flavonoids

24	Luteolin-6,8-di-C-glucoside	C27H30O16	Flavonoids
25	Eupatilin 3-glucoside	C24H26O12	Flavonoids
26	Verbascoside	C29H36O15	Phenolic acids
27	N-Acetyl-DL-tryptophan	C15H18N2O4	Amino acids and derivatives
28	Quillaic acid	C30H46O5	Phenolic acids
29	3-Hydroxy-4-isopropylbenzylalcohol 3-glucoside	C16H24O7	Phenolic acids
30	Isorhamnetin 3-O-β-(2''-O-acetyl-β-D-glucuronide)	C24H22O14	Flavonoids
31	(+)-Syringaresinol	C22H26O8	Lignans and Coumarins
32	Ayapin	C11H8O3	Lignans and Coumarins
33	2,4,6,4'-Tetrahydroxy-stilbene-2-O-D-glucopyranoside	C20H22O9	Others
34	Terpineol monO-glucoside	C26H32O11	Lignans and Coumarins
35	Trans,trans-Muconic acid	C6H6O4	Organic acids
36	Uridine 5′-diphosphate	C9H14N2O12P2	Nucleotides and derivatives
37	Luteolin 7-O-glucoside(Cynaroside)	C21H20O11	Flavonoids
38	Hesperetin 5-O-glucoside	C22H24O11	Flavonoids
39	2'-Deoxycytidine-5'-monophosphate	C9H14N3O7P	Nucleotides and derivatives
40	Kaempferol 3-O-rutinoside(Nicotiflorin)	C27H30O15	Flavonoids
41	Xanthine	C5H4N4O2	Nucleotides and derivatives
42	LysoPC 16:0(2n isomer)	C24H50NO7P	Lipids
43	Luteolin C-hexoside	C21H20O11	Flavonoids
44	Apigenin C-hexosyl-O-rutinoside	C33H40O19	Flavonoids
45	Hesperetin O-malonylhexoside	C25H26O14	Flavonoids
46	Disinapoyl hexoside	C28H34O16	Phenolic acids
47	2,5-Dihydroxy benzoic acid O-hexside	C28H32O14	Phenolic acids
48	9-HOTrE	C13H16O9	Lipids

49	3,4-Dimethoxycinnamic acid	$C_{18}H_{30}O_3$	Phenolic acids
50	Acetyltryptophan	$C_{11}H_{12}O_4$	Amino acids and derivatives
51	MAG(18:3)isomer1	$C_{13}H_{14}N_2O_3$	Lipids
52	MAG(18:1)isomer1	$C_{21}H_{36}O_4$	Lipids
53	LysoPC 15:0	$C_{21}H_{40}O_4$	Lipids
54	Cocamidopropyl βine	$C_{23}H_{48}NO_7P$	Alkaloids
55	MAG(18:3)isomer4	$C_{19}H_{38}N_2O_3$	Lipids
56	MAG(18:3)isomer3	$C_{21}H_{36}O_4$	Lipids
57	N'-Feruloyl putrescine	$C_{21}H_{36}O_4$	Alkaloids
58	LysoPE 16:0	$C_{14}H_{20}N_2O_3$	Lipids
59	LysoPC 18:3(2n isomer)	$C_{21}H_{44}NO_7P$	Lipids
60	LysoPE 14:0	$C_{26}H_{48}NO_7P$	Lipids
61	LysoPC 16:0	$C_{19}H_{40}NO_7P$	Lipids
62	LysoPC 18:3	$C_{24}H_{50}NO_7P$	Lipids
63	Tricin O-malonylhexoside	$C_{26}H_{48}NO_7P$	Flavonoids
64	Tricin 7-O-hexosyl-O-hexoside	$C_{26}H_{26}O_{15}$	Flavonoids
65	Apigenin 8-C-pentoside	$C_{29}H_{34}O_{17}$	Flavonoids
66	8-C-Hexosyl-apigenin O-feruloylhexoside	$C_{20}H_{18}O_9$	Flavonoids
67	6-C-Hexosyl luteolin O-pentoside	$C_{37}H_{38}O_{18}$	Flavonoids
68	C-Hexosyl-luteolin C-pentoside	$C_{26}H_{28}O_{15}$	Flavonoids
69	8-C-Hexosyl-hesperetin O-hexoside	$C_{26}H_{28}O_{15}$	Flavonoids
70	Hesperetin C-hexosyl-O-hexosyl-O-hexoside	$C_{28}H_{34}O_{16}$	Flavonoids
71	Luteolin O-hexosyl-O-pentoside	$C_{34}H_{44}O_{21}$	Flavonoids
72	N-Sinapoyl putrescine	$C_{26}H_{28}O_{15}$	Alkaloids
73	N-p-Coumaroyl putrescine	$C_{15}H_{22}N_2O_4$	Alkaloids

74	12-Hydroxydodecanoic acid	C13H18N2O2	Lipids
75	Turanose	C12H24O3	Others
76	Sinapic acid	C12H21O11Na	Phenolic acids
77	Sinapic acid	C11H12O5	Phenolic acids
78	Dopamine hydrochloride	C8H11NO2	Alkaloids
79	Peonidin 3-O-glucoside	C22H23O11+	Flavonoids
80	Schaftoside	C26H28O14	Flavonoids
81	HoMoorientin	C21H20O11	Flavonoids
82	Trans-ferulic acid	C10H10O4	Phenolic acids
83	Orientin	C21H20O11	Flavonoids
84	Isoschaftoside	C26H28O14	Flavonoids
85	Spiraeoside	C21H20O12	Flavonoids
86	(S)-2-(4-Aminobutanamido)-3-(1-methyl-1H-imidazol-5-yl)propanoic acid	C10H16N4O3	Organic acids
87	4-Hydroxybenzoic acid	C7H6O3	Phenolic acids
88	N-Acetyl-5-hydroxytryptamine	C12H14N2O2	Alkaloids
89	Cis-10-Heptadecenoic Acid	C17H32O2	Lipids
90	Palmitoleic Acid	C16H30O2	Lipids
91	Anchoic Acid	C9H16O4	Organic acids
92	1-Stearoyl-sn-glycero-3-phosphocholine	C26H54NO7P	Lipids
93	N-(3-Indolylacetyl)-L-alanine	C13H14N2O3	Amino acids and derivatives
94	Pinoresinol	C20H22O6	Lignans and Coumarins
95	Kaempferol glc-rha	C27H30O15	Flavonoids
96	Narirutin 4'-glucoside	C33H42O19	Flavonoids
97	Kaempferol 3-O-β-d-(6''-O-(E)-p-coumaroyl)glucopyranoside	C30H26O13	Flavonoids

98	p-Coumaroylmalic acid	C13H12O7	Phenolic acids
99	Apigenin 8-C-α-L-Arabinopyranoside	C20H18O9	Flavonoids
100	Kaempferol-3-O-neohesperidoside	C27H30O15	Flavonoids
101	Kaempferol-3,7-di-O-β-D-glucopyranoside	C27H30O16	Flavonoids
102	PC(18:2/18:3+O3)	C44H78NO11P	Lipids
103	PE(oxo-11:0/16:0)	C32H62NO9P	Lipids
104	PC(oxo-11:0/18:2)	C37H68NO9P	Lipids
105	LysoPG(16:0)	C22H45O9P	Lipids
106	Salidroside	C14H20O7	Phenolic acids
107	Glucosyloxybenzoic acid	C13H16O8	Phenolic acids
108	Luteolin-7-O-β-D-gentiobioside	C27H30O16	Flavonoids
109	Cymbinodin A	C15H10O4	Quinones
110	3,4-Dihydroxybenzaldehyde	C7H6O3	Phenolic acids
111	6-Hydroxy-7-methoxy-coumarin	C10H8O4	Lignans and Coumarins
112	Kaempferol-O-Pentoside-O-hexoside	C26H28O15	Flavonoids
113	Isorhamnetin-O-rutinoside	C28H32O16	Flavonoids
114	Isorhamnetin-O-Hexoside-O-Hexoside	C28H32O17	Flavonoids
115	6-Hydroxyluteolin 5-glucoside	C21H20O12	Flavonoids
116	Quercetin-O-rutinoside-hexose	C33H40O21	Flavonoids

Table S2. Differential metabolites between PB and PC.

No.	Compounds	Formula	Class	VIP	Log2FC	Type
1	Isochrysoeriol C-hexosyl-O-hexoside	C28H32O16	Flavonoids	1.37E+00	-1.14E+00	down
2	Iso-8-C-hexosyl-luteolin O-hexoside	C27H30O16	Flavonoids	1.60E+00	-2.06E+00	down
3	6-Hydroxykaempferol-3,7,6-O-triglycoside	C33H40O22	Flavonoids	1.24E+00	-1.09E+00	down
4	6-Hydroxykaempferol-7,6-O-Diglucoside	C27H30O17	Flavonoids	1.33E+00	-1.41E+00	down

5	6-Hydroxykaempferol-3,6-O-Diglucoside	C27H30O17	Flavonoids	1.79E+00	-2.12E+00	down
6	Hesperetin C-malonylhexoside	C26H28O13	Flavonoids	1.42E+00	-4.20E+00	down
7	Quercetin 3,7-bis-O-β-D-glucoside	C27H30O17	Flavonoids	1.08E+00	-1.05E+00	down
8	Luteolin-7,3'-Di-O-β-D-Glucoside	C27H30O16	Flavonoids	1.61E+00	-2.02E+00	down
9	liquiritigenin-7,4-diglucoside	C27H32O14	Flavonoids	1.01E+00	-1.65E+00	down
10	Verbascoside	C29H36O15	Phenolic acids	1.41E+00	-1.36E+00	down
11	3-Hydroxy-4-isopropylbenzylalcohol 3-glucoside	C16H24O7	Phenolic acids	1.52E+00	-1.43E+00	down
12	(+)-Syringaresinol	C22H26O8	Lignans and Coumarins	1.24E+00	-1.34E+00	down
13	Terpineol monO-glucoside	C26H32O11	Lignans and Coumarins	1.12E+00	-1.17E+00	down
14	Luteolin 7-O-glucoside(Cynaroside)	C21H20O11	Flavonoids	1.98E+00	-1.98E+00	down
15	Hesperetin 5-O-glucoside	C22H24O11	Flavonoids	1.32E+00	-1.34E+00	down
16	Xanthine	C5H4N4O2	Nucleotides and derivatives	1.06E+00	-2.46E+00	down
17	Hesperetin O-malonylhexoside	C25H26O14	Flavonoids	1.97E+00	-1.38E+00	down
18	3,4-Dimethoxycinnamic acid	C11H12O4	Phenolic acids	1.69E+00	-1.41E+00	down
19	MAG(18:1)isomer1	C21H40O4	Lipids	1.82E+00	-2.13E+00	down
20	Hesperetin C-hexosyl-O-hexosyl-O-hexoside	C34H44O21	Flavonoids	1.20E+00	-1.14E+00	down
21	Spiraeoside	C21H20O12	Flavonoids	1.04E+00	-1.34E+00	down
22	4-Hydroxybenzoic acid	C7H6O3	Phenolic acids	1.74E+00	-1.04E+00	down
23	Cis-10-Heptadecenoic Acid	C17H32O2	Lipids	1.65E+00	-1.33E+00	down
24	Palmitoleic Acid	C16H30O2	Lipids	1.77E+00	-1.07E+00	down
25	Narirutin 4'-glucoside	C33H42O19	Flavonoids	1.86E+00	-1.33E+00	down
26	Kaempferol-3,7-di-O-β-D-glucopyranoside	C27H30O16	Flavonoids	1.72E+00	-2.70E+00	down
27	Luteolin-7-O-β-D-gentiobioside	C27H30O16	Flavonoids	1.73E+00	-2.65E+00	down
28	Cymbinodin A	C15H10O4	Quinones	1.33E+00	-2.39E+00	down

29	3,4-Dihydroxybenzaldehyde	C7H6O3	Phenolic acids	1.67E+00	-1.03E+00	down
30	6-Hydroxy-7-methoxy-coumarin	C10H8O4	Lignans and Coumarins	1.16E+00	-1.32E+00	down
31	Kaempferol-O-Pentoside-O-hexoside	C26H28O15	Flavonoids	1.14E+00	-1.94E+00	down
32	Isorhamnetin-O-rutinoside	C28H32O16	Flavonoids	1.38E+00	-1.17E+00	down
33	Isorhamnetin-O-Hexoside-O-Hexoside	C28H32O17	Flavonoids	1.50E+00	-1.00E+00	down
34	6-Hydroxyluteolin 5-glucoside	C21H20O12	Flavonoids	1.28E+00	-1.02E+00	down
35	Isosinapic acid-hexoside	C17H22O10	Phenolic acids	1.40E+00	1.15E+00	up
36	LysoPC(18:0)	C26H54NO7P	Lipids	1.66E+00	1.03E+00	up
37	PC(18:2)isomer	C26H51NO7P+	Lipids	1.65E+00	1.05E+00	up
38	Apigenin-6-C-β-D-xyloside-8-C-β-Darabinoside	C25H26O13	Flavonoids	1.64E+00	1.15E+00	up
39	Vitexin-2-O-D-glucopyranoside	C27H30O15	Flavonoids	1.61E+00	1.01E+00	up
40	Luteolin-6-C-2-glucuronylglucoside	C27H28O17	Flavonoids	1.92E+00	1.23E+00	up
41	Chysoeriol-6,8-di-C-glucoside	C28H32O16	Flavonoids	1.90E+00	1.18E+00	up
42	Eupatilin 3-glucoside	C24H26O12	Flavonoids	1.73E+00	1.06E+00	up
43	2,4,6,4'-Tetrahydroxy-stilbene-2-O-D-glucopyranoside	C20H22O9	Others	1.67E+00	1.65E+00	up
44	Uridine 5′-diphosphate	C9H14N2O12P2	Nucleotides and derivatives	1.64E+00	1.29E+00	up
45	LysoPC 16:0(2n isomer)	C24H50NO7P	Lipids	1.77E+00	1.25E+00	up
46	Apigenin C-hexosyl-O-rutinoside	C33H40O19	Flavonoids	1.41E+00	1.33E+00	up
47	9-HOTrE	C18H30O3	Lipids	1.33E+00	1.20E+00	up
48	MAG(18:3)isomer1	C21H36O4	Lipids	1.70E+00	1.14E+00	up
49	LysoPC 15:0	C23H48NO7P	Lipids	1.50E+00	1.12E+00	up
50	MAG(18:3)isomer4	C21H36O4	Lipids	1.85E+00	1.09E+00	up
51	N'-Feruloyl putrescine	C14H20N2O3	Alkaloids	1.86E+00	2.46E+00	up
52	LysoPC 18:3(2n isomer)	C26H48NO7P	Lipids	1.81E+00	2.07E+00	up

53	LysoPC 16:0	C24H50NO7P	Lipids	1.74E+00	1.25E+00	up
54	LysoPC 18:3	C26H48NO7P	Lipids	1.83E+00	2.14E+00	up
55	Tricin O-malonylhexoside	C26H26O15	Flavonoids	1.66E+00	1.14E+00	up
56	Tricin 7-O-hexosyl-O-hexoside	C29H34O17	Flavonoids	1.16E+00	1.03E+00	up
57	8-C-Hexosyl-apigenin O-feruloylhexoside	C37H38O18	Flavonoids	1.46E+00	1.07E+00	up
58	N-Sinapoyl putrescine	C15H22N2O4	Alkaloids	1.31E+00	2.04E+00	up
59	N-p-Coumaroyl putrescine	C13H18N2O2	Alkaloids	1.79E+00	2.55E+00	up
60	Sinapic acid	C11H12O5	Phenolic acids	1.94E+00	2.04E+00	up
61	Schaftoside	C26H28O14	Flavonoids	1.94E+00	1.57E+00	up
62	Trans-ferulic acid	C10H10O4	Phenolic acids	1.37E+00	1.01E+00	up
63	Isoschaftoside	C26H28O14	Flavonoids	1.74E+00	1.40E+00	up
64	N-Acetyl-5-hydroxytryptamine	C12H14N2O2	Alkaloids	1.54E+00	1.05E+00	up
65	Anchoic Acid	C9H16O4	Organic acids	1.02E+00	1.04E+00	up
66	PE(oxo-11:0/16:0)	C32H62NO9P	Lipids	1.51E+00	2.28E+00	up
67	PC(oxo-11:0/18:2)	C37H68NO9P	Lipids	1.35E+00	1.77E+00	up
68	LysoPG(16:0)	C22H45O9P	Lipids	1.76E+00	1.11E+00	up

Table S3. Differential metabolites between PB and CC.

**No.**	**Compounds**	**Formula**	**Class**	**VIP**	**Log2FC**	**Type**
1	Iso-8-C-hexosyl-luteolin O-hexoside	C27H30O16	Flavonoids	1.74E+00	-1.37E+00	down
2	Isoluteolin-6,8-di-C-glucoside	C27H30O16	Flavonoids	1.21E+00	-1.22E+00	down
3	Salicylic acid O-glycoside	C13H16O8	Phenolic acids	1.26E+00	-1.12E+00	down
4	6-Hydroxykaempferol-3,7,6-O-triglycoside	C33H40O22	Flavonoids	1.30E+00	-1.01E+00	down
5	6-Hydroxykaempferol-7,6-O-Diglucoside	C27H30O17	Flavonoids	1.55E+00	-1.44E+00	down
6	6-Hydroxykaempferol-3,6-O-Diglucoside	C27H30O17	Flavonoids	1.77E+00	-1.48E+00	down
7	6-Hydroxykaempferol-7-O-glucoside	C21H20O12	Flavonoids	1.04E+00	-1.17E+00	down

8	Caffeoylcholine 6-glucoside	C20H30NO9+	Alkaloids	1.83E+00	-1.57E+00	down
9	Hesperetin C-malonylhexoside	C26H28O13	Flavonoids	1.57E+00	-4.04E+00	down
10	Quercetin 3,7-bis-O-β-D-glucoside	C27H30O17	Flavonoids	1.40E+00	-1.12E+00	down
11	Luteolin-7,3'-Di-O-β-D-Glucoside	C27H30O16	Flavonoids	1.82E+00	-1.36E+00	down
12	4-Caffeoylquinic acid	C16H18O9	Phenolic acids	1.06E+00	-1.43E+00	down
13	Luteolin-6,8-di-C-glucoside	C27H30O16	Flavonoids	1.54E+00	-1.17E+00	down
14	(+)-Syringaresinol	C22H26O8	Lignans and Coumarins	1.18E+00	-1.01E+00	down
15	Luteolin C-hexoside	C21H20O11	Flavonoids	1.75E+00	-2.22E+00	down
16	Disinapoyl hexoside	C28H32O14	Phenolic acids	1.30E+00	-1.29E+00	down
17	2,5-Dihydroxy benzoic acid O-hexside	C13H16O9	Phenolic acids	2.16E+00	-1.16E+00	down
18	MAG(18:1)isomer1	C21H40O4	Lipids	1.83E+00	-1.54E+00	down
19	Cocamidopropyl βine	C19H38N2O3	Alkaloids	2.21E+00	-2.62E+00	down
20	6-C-Hexosyl luteolin O-pentoside	C26H28O15	Flavonoids	1.41E+00	-2.52E+00	down
21	8-C-Hexosyl-hesperetin O-hexoside	C28H34O16	Flavonoids	1.47E+00	-1.16E+00	down
22	Hesperetin C-hexosyl-O-hexosyl-O-hexoside	C34H44O21	Flavonoids	1.31E+00	-1.11E+00	down
23	Dopamine hydrochloride	C8H11NO2	Alkaloids	2.08E+00	-1.13E+00	down
24	HoMoorientin	C21H20O11	Flavonoids	1.91E+00	-1.33E+00	down
25	Orientin	C21H20O11	Flavonoids	1.83E+00	-2.00E+00	down
26	(S)-2-(4-Aminobutanamido)-3-(1-methyl-1H-imidazol-5-yl)propanoic acid	C10H16N4O3	Organic acids	2.00E+00	-3.18E+00	down
27	PC(18:2/18:3+O3)	C44H78NO11P	Lipids	1.54E+00	-1.01E+00	down
28	Salidroside	C14H20O7	Phenolic acids	1.30E+00	-1.11E+00	down
29	Glucosyloxybenzoic acid	C13H16O8	Phenolic acids	1.30E+00	-1.10E+00	down
30	Quercetin-O-rutinoside-hexose	C33H40O21	Flavonoids	1.07E+00	-1.17E+00	down
31	Apigenin-6-C-glucose-8-xylcose	C26H28O14	Flavonoids	1.32E+00	1.11E+00	up

32	N-Acetyl-DL-tryptophan	C15H18N2O4	Amino acids and derivatives	1.26E+00	1.40E+00	up
33	Quillaic acid	C30H46O5	Phenolic acids	1.56E+00	1.18E+00	up
34	Isorhamnetin 3-O-β-(2''-O-acetyl-β-D-glucuronide)	C24H22O14	Flavonoids	1.55E+00	4.41E+00	up
35	Ayapin	C11H8O3	Lignans and Coumarins	1.55E+00	1.09E+00	up
36	Trans,trans-Muconic acid	C6H6O4	Organic acids	2.06E+00	1.87E+00	up
37	Uridine 5′-diphosphate	C9H14N2O12P2	Nucleotides and derivatives	1.91E+00	1.33E+00	up
38	2'-Deoxycytidine-5'-monophosphate	C9H14N3O7P	Nucleotides and derivatives	1.10E+00	1.13E+00	up
39	Acetyltryptophan	C13H14N2O3	Amino acids and derivatives	1.30E+00	1.27E+00	up
40	MAG(18:3)isomer3	C21H36O4	Lipids	1.63E+00	1.15E+00	up
41	LysoPC 18:3(2n isomer)	C26H48NO7P	Lipids	1.22E+00	1.06E+00	up
42	LysoPC 18:3	C26H48NO7P	Lipids	1.21E+00	1.03E+00	up
43	Apigenin 8-C-pentoside	C20H18O9	Flavonoids	1.93E+00	2.31E+00	up
44	12-Hydroxydodecanoic acid	C12H24O3	Lipids	1.52E+00	1.19E+00	up
45	Turanose	C12H21O11Na	Others	2.03E+00	1.58E+00	up
46	Sinapic acid	C11H12O5	Phenolic acids	1.04E+00	1.01E+00	up
47	Anchoic Acid	C9H16O4	Organic acids	1.53E+00	1.05E+00	up
48	N-(3-Indolylacetyl)-L-alanine	C13H14N2O3	Amino acids and derivatives	1.22E+00	1.05E+00	up
49	Pinoresinol	C20H22O6	Lignans and Coumarins	1.06E+00	1.05E+00	up
50	p-Coumaroylmalic acid	C13H12O7	Phenolic acids	1.87E+00	3.98E+00	up
51	Apigenin 8-C-α-L-Arabinopyranoside	C20H18O9	Flavonoids	1.23E+00	2.38E+00	up

Table S4. Differential metabolites between PC and CC.

No.	Compounds	Formula	Class I	VIP	Log2FC	Type
1	Pinoresinol-acetylglucose	C28H34O12	Lignans and Coumarins	1.38E+00	-2.48E+00	down
2	liquiritigenin-7,4-diglucoside	C27H32O14	Flavonoids	1.32E+00	-1.43E+00	down
3	Verbascoside	C29H36O15	Phenolic acids	1.55E+00	-1.32E+00	down
4	Isorhamnetin 3-O-β-(2''-O-acetyl-β-D-glucuronide)	C24H22O14	Flavonoids	1.41E+00	-2.43E+00	down
5	Terpineol monO-glucoside	C26H32O11	Lignans and Coumarins	1.70E+00	-2.11E+00	down
6	Trans,trans-Muconic acid	C6H6O4	Organic acids	1.87E+00	-1.62E+00	down
7	Luteolin 7-O-glucoside(Cynaroside)	C21H20O11	Flavonoids	1.95E+00	-1.62E+00	down
8	Kaempferol 3-O-rutinoside(Nicotiflorin)	C27H30O15	Flavonoids	1.63E+00	-2.32E+00	down
9	3,4-Dimethoxycinnamic acid	C11H12O4	Phenolic acids	1.99E+00	-1.22E+00	down
10	Luteolin O-hexosyl-O-pentoside	C26H28O15	Flavonoids	1.30E+00	-1.37E+00	down
11	12-Hydroxydodecanoic acid	C12H24O3	Lipids	1.69E+00	-1.65E+00	down
12	Pinoresinol	C20H22O6	Lignans and Coumarins	1.43E+00	-1.64E+00	down
13	Kaempferol glc-rha	C27H30O15	Flavonoids	1.62E+00	-2.22E+00	down
14	Kaempferol 3-O-β-d-(6''-O-(E)-p-coumaroyl)glucopyranoside	C30H26O13	Flavonoids	1.58E+00	-2.09E+00	down
15	p-Coumaroylmalic acid	C13H12O7	Phenolic acids	1.32E+00	-2.58E+00	down
16	Kaempferol-3-O-neohesperidoside	C27H30O15	Flavonoids	1.58E+00	-2.15E+00	down
17	Kaempferol-3,7-di-O-β-D-glucopyranoside	C27H30O16	Flavonoids	1.70E+00	-2.05E+00	down
18	Luteolin-7-O-β-D-gentiobioside	C27H30O16	Flavonoids	1.65E+00	-2.03E+00	down
19	Cymbinodin A	C15H10O4	Quinones	1.37E+00	-1.17E+00	down
20	6-Hydroxy-7-methoxy-coumarin	C10H8O4	Lignans and	1.26E+00	-1.08E+00	down

			Coumarins			
21	Kaempferol-O-Pentoside-O-hexoside	C26H28O15	Flavonoids	1.36E+00	-1.56E+00	down
22	Isoluteolin-6,8-di-C-glucoside	C27H30O16	Flavonoids	2.07E+00	1.67E+00	up
23	Salicylic acid O-glycoside	C13H16O8	Phenolic acids	1.69E+00	1.16E+00	up
24	LysoPC(18:0)	C26H54NO7P	Lipids	1.86E+00	1.21E+00	up
25	Luteolin-6,8-di-C-glucoside	C27H30O16	Flavonoids	2.00E+00	1.43E+00	up
26	2,4,6,4'-Tetrahydroxy-stilbene-2-O-D-glucopyranoside	C20H22O9	Others	1.34E+00	1.00E+00	up
27	LysoPC 16:0(2n isomer)	C24H50NO7P	Lipids	1.85E+00	1.30E+00	up
28	Luteolin C-hexoside	C21H20O11	Flavonoids	2.04E+00	1.76E+00	up
29	Apigenin C-hexosyl-O-rutinoside	C33H40O19	Flavonoids	1.17E+00	1.52E+00	up
30	Hesperetin O-hexosyl-O-hexoside	C28H34O16	Flavonoids	1.61E+00	1.24E+00	up
31	MAG(18:3)isomer1	C21H36O4	Lipids	1.99E+00	1.15E+00	up
32	LysoPC 15:0	C23H48NO7P	Lipids	1.81E+00	1.20E+00	up
33	Cocamidopropyl βine	C19H38N2O3	Alkaloids	1.97E+00	1.64E+00	up
34	N'-Feruloyl putrescine	C14H20N2O3	Alkaloids	2.03E+00	1.64E+00	up
35	LysoPE 16:0	C21H44NO7P	Lipids	1.98E+00	1.03E+00	up
36	LysoPC 18:3(2n isomer)	C26H48NO7P	Lipids	1.48E+00	1.00E+00	up
37	LysoPE 14:0	C19H40NO7P	Lipids	2.06E+00	1.21E+00	up
38	LysoPC 16:0	C24H50NO7P	Lipids	1.84E+00	1.33E+00	up
39	LysoPC 18:3	C26H48NO7P	Lipids	1.55E+00	1.11E+00	up
40	Tricin 7-O-hexosyl-O-hexoside	C29H34O17	Flavonoids	1.24E+00	1.08E+00	up
41	8-C-Hexosyl-apigenin O-feruloylhexoside	C37H38O18	Flavonoids	1.78E+00	1.61E+00	up
42	6-C-Hexosyl luteolin O-pentoside	C26H28O15	Flavonoids	1.88E+00	3.17E+00	up
43	C-Hexosyl-luteolin C-pentoside	C26H28O15	Flavonoids	1.68E+00	1.04E+00	up
44	N-Sinapoyl putrescine	C15H22N2O4	Alkaloids	1.92E+00	1.34E+00	up

45	N-p-Coumaroyl putrescine	C13H18N2O2	Alkaloids	1.89E+00	1.63E+00	up
46	Sinapic acid	C11H12O5	Phenolic acids	1.47E+00	1.03E+00	up
47	Peonidin 3-O-glucoside	C22H23O11+	Flavonoids	1.37E+00	1.59E+00	up
48	HoMoorientin	C21H20O11	Flavonoids	1.88E+00	1.12E+00	up
49	Trans-ferulic acid	C10H10O4	Phenolic acids	1.70E+00	1.10E+00	up
50	Orientin	C21H20O11	Flavonoids	2.08E+00	1.58E+00	up
51	(S)-2-(4-Aminobutanamido)-3-(1-methyl-1H-imidazol-5-yl)propanoic acid	C10H16N4O3	Organic acids	1.77E+00	2.81E+00	up
52	1-Stearoyl-sn-glycero-3-phosphocholine	C26H54NO7P	Lipids	1.82E+00	1.16E+00	up
53	PC(18:2/18:3+O3)	C44H78NO11P	Lipids	1.26E+00	1.41E+00	up
54	PE(oxo-11:0/16:0)	C32H62NO9P	Lipids	1.40E+00	1.58E+00	up
55	PC(oxo-11:0/18:2)	C37H68NO9P	Lipids	1.25E+00	1.38E+00	up
56	Salidroside	C14H20O7	Phenolic acids	1.68E+00	1.24E+00	up
57	Glucosyloxybenzoic acid	C13H16O8	Phenolic acids	1.72E+00	1.23E+00	up
